# Currarino syndrome in an adult presenting with a presacral abscess: a case report

**DOI:** 10.1186/1752-1947-8-77

**Published:** 2014-02-27

**Authors:** Masatoshi Shoji, Naomi Nojima, Akemi Yoshikawa, Wataru Fukushima, Naotaka Kadoya, Hisashi Hirosawa, Ryohei Izumi

**Affiliations:** 1 Department of Gastroenterological Surgery, Division of Cancer Medicine, Graduate School of Medical Science, Kanazawa University, 13-1 Takara-machi, Kanazawa, Ishikawa 920-8641, Japan; 2 Department of Surgery, Toyama City Hospital, Toyama 939-8511, Japan; 3 Department of Surgery, Toyama Rosai Hospital, Toyama 937-0042, Japan

**Keywords:** Adult, Currarino syndrome, Epidermal cyst, Presacral abscess

## Abstract

**Introduction:**

Currarino syndrome (Currarino triad) was described in 1981 as a triad syndrome with a common embryogenesis in infants and with three characteristics: anorectal stenosis, a defect in the sacral bone, and a presacral mass. We describe here an unusual case of Currarino syndrome in an adult presenting with a presacral abscess but no meningitis.

**Case presentation:**

A 32-year-old Japanese man presented with fever, arthralgia and buttock pain. A digital rectal examination showed mild rectal stenosis with local warmth and tenderness in the posterior wall of his rectum. Computed tomography showed a scimitar-shaped deformity of his sacrum and an 8cm presacral mass, which continued to a pedicle of his deformed sacrum. This was diagnosed as Currarino syndrome with a presacral abscess. The abscess was drained by a perianal approach with our patient treated with antibiotics. His symptoms soon disappeared. After three months, an excision was performed through a posterior sagittal approach. His postoperative course was uneventful and he was discharged 10 days after surgery. A histopathological examination revealed an infected epidermoid cyst. He has been free from recurrence as of four years and six months after surgery.

**Conclusions:**

We report a case of Currarino syndrome in an adult who presented with a presacral abscess but no meningitis. Abscess drainage followed by radical surgery resulted in a successful outcome.

## Introduction

Currarino syndrome (CS), also known as Currarino triad, was reported as a syndrome complex with a common embryogenesis by Currarino *et al.* in 1981 [1]. It consists of congenital caudal anomalies with three main characteristics: anorectal stenosis, sacral defect, and presacral mass. CS is an autosomal dominant disorder, and the result of mutations in the homeobox gene *HLXB9* on chromosome 7 [2-4]. These mutations can be incidental. About 270 patients with this disorder have been reported up to the year 2012 [5]. The precise incidence of CS is not well known because of various phenotypes and clinical presentations. The presacral mass has been reported to be an anterior sacral meningocele in 60% of patients, a teratoma in 25%, and other tumors in the remaining 15% of patients [6]. The presence of life-threatening complications aids the recognition and diagnosis of CS. However, there is serious concern in undiagnosed patients who are clinically asymptomatic because of the risk of complications such as meningitis, neurological injury and even malignancy. Crucial complications associated with the congenital caudal anomalies present in CS sometimes need surgical management. The presence of clinical variations can lead to diagnostic difficulties. A careful radiological assessment is important to select the most suitable treatment.

We report an unusual case of CS in an adult presenting with a presacral abscess. There was an early suspicion of CS, and a multidisciplinary assessment resulted in successful treatment.

## Case presentation

A 32-year-old Japanese man was referred to our hospital complaining of fever, arthralgia and buttock pain. His birth history was unremarkable. He had a past history of bronchial asthma and gastric ulcer without constipation. On admission, his temperature was 40.0°C. An abdominal examination showed no tenderness, no distention and no palpable mass. A neurological examination showed no contributory factors. The anal location and tonus were normal. A digital rectal examination demonstrated mild rectal stenosis with local warmth and tenderness in the posterior wall of his rectum.

**Figure 1:**
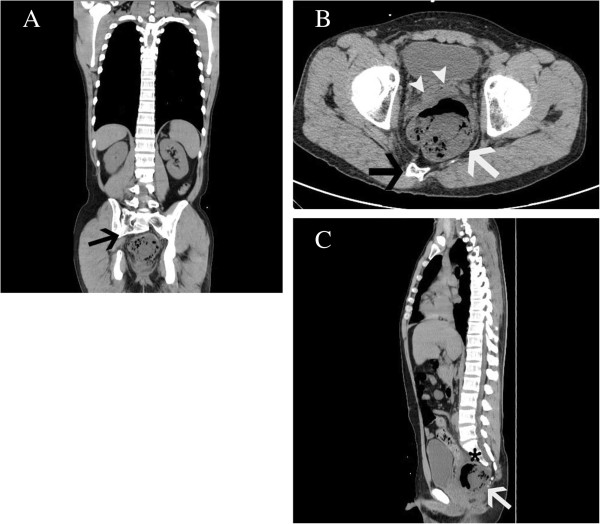
**Computed tomography imaging findings. (A)** A scimitar-shaped deformity of the sacrum (black arrow). **(B)** An 8cm presacral mass (white arrow) containing air displaced the rectum (white arrow heads) ventrally. **(C)** The mass appeared to continue to the spinal canal through the anterior wall of the sacrum (asterisk).

**Figure 2:**
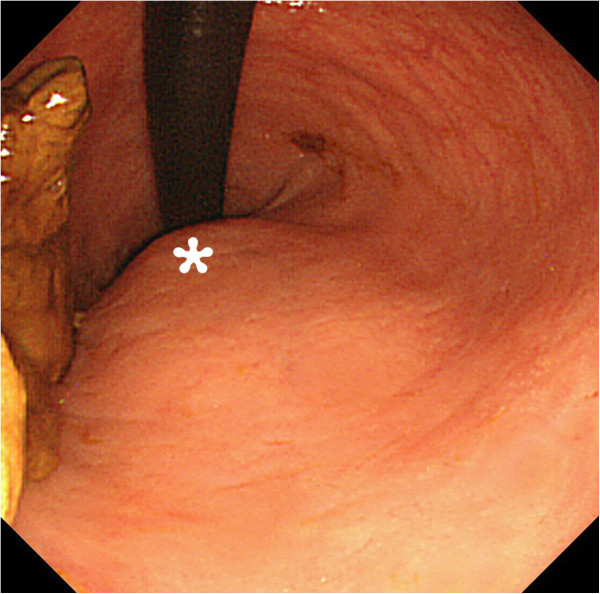
**Sigmoidoscopy findings.** The posterior wall of the rectum was compressed extramurally (asterisk).

Laboratory studies revealed a white blood cell count of 18,000 cells/μL and C-reactive protein of 26.11mg/dL. Results of a urine analysis were normal. Computed tomography (CT) showed a scimitar-shaped deformity of his sacrum. An 8cm presacral mass containing air displaced his rectum ventrally and appeared to continue to his spinal canal through the anterior wall of his sacrum (Figure 1). Sigmoidoscopy showed extramural compression on the posterior of his rectum (Figure 2). We therefore diagnosed our patient with CS with a presacral abscess.

Immediate attention was given by draining the perianal abscess. The eruption of yellowish purulent fluid and keratinous debris were observed, and an infected epidermoid cyst was suspected. The pus yielded a culture of *Streptococcus anginosus* and *Bacteroides fragilis*. We treated him with antibiotics and his symptoms improved within a few days.

**Figure 3:**
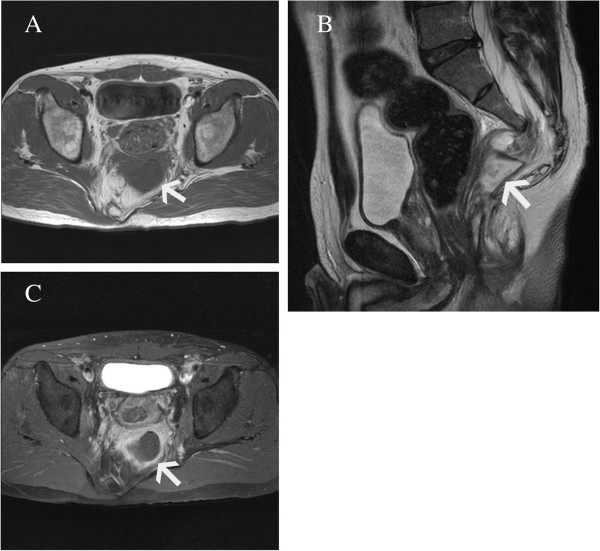
**Magnetic resonance imaging findings. (A)** Axial T1-weighted image showing a low density of the presacral mass (white arrow). **(B)** A tethered cord could not be revealed on a sagittal T2-weighted image. **(C)** The mass was not enhanced on contrast-enhanced magnetic resonance imaging. The cystic wall was thick, surrounding a fuzzy tissue.

**Figure 4:**
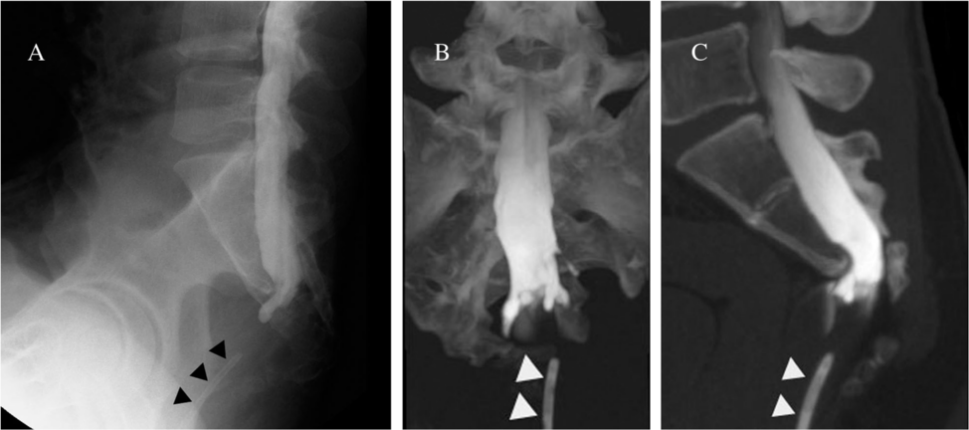
**Myelography and postmyelography computed tomography findings. (A)** Sagittal myelography showed no apparent communication between the presacral mass and the thecal sac. **(B, C)** Coronal and sagittal postmyelography computed tomography demonstrated that the terminus of the thecal sac formed some processes. The drain (black (A) and white (B, C) triangles) was placed via a perianal insertion.

**Figure 5:**
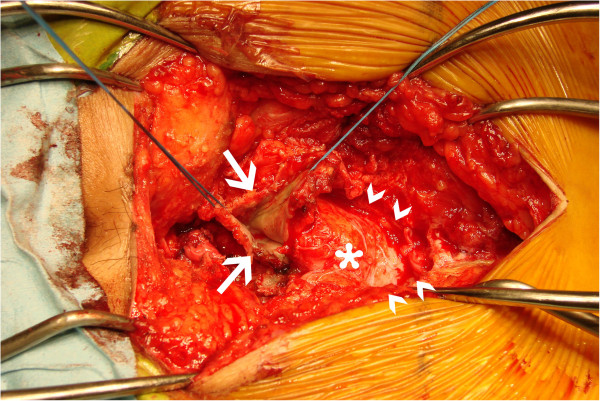
**Intraoperative phase.** Exploration through a posterior sagittal approach demonstrated a silvery white epidermoid tumor (white arrow) occupying the presacral space. The tumor was poorly circumscribed and firmly adhered to surrounding tissues containing bilateral S3 sacral nerve roots (white arrow heads) and dura (asterisk).

Later, magnetic resonance imaging (MRI) showed the presacral mass with the same imaging characteristics as an infected epidermoid cyst (Figure 3). Myelography and postmyelography CT showed no apparent communication between the presacral mass and the thecal sac (Figure 4). Results of an analysis of his cerebrospinal fluid were normal, and culture of the fluid identified no organisms. A radical operation was performed three months after abscess drainage. An exploration using a posterior sagittal approach demonstrated a silvery white epidermoid tumor occupying the presacral space. Purulent fluid was not observed. The tumor was poorly circumscribed and firmly adhered to surrounding tissues containing S3 sacral nerve roots and dura (Figure 5). The tissues were sharply dissected and excised without injuring the nerves. His rectum was intact.

Histopathology confirmed our diagnosis of an infected epidermoid cyst. The cystic wall consisted of stratified squamous epithelium. Our patient’s postoperative course was uneventful, and he was discharged 10 days after surgery. The *HLXB9* mutation was not evaluated because he declined a gene test. On a follow-up appointment at four years and six months, our patient was doing well without any neurological deficit and with normal defecation.

## Discussion

To date, approximately 300 cases of CS have been reported in the English literature [4,7,8]. The majority of patients with CS have autosomal dominant inheritance of mutations in the homeobox gene *HLXB9*, which encodes the nuclear protein HB9. However, a genotype-phenotype correlation has not been reported [6]. Kim *et al.* reported variable clinical and imaging features of CS in three siblings with a *HLXB9* gene mutation [9]. In fact, CS includes a variety of clinical expressions. Many patients show an asymptomatic hemisacrum without any other life-threatening complications; one third of patients with CS are asymptomatic and may be diagnosed late in life [4]. The condition shows a gender bias, with a male to female occurrence ratio of two to one. The most common symptom is chronic constipation in childhood. The frequency of constipation in adulthood is lower than in childhood [10]. Other clinical features are a presacral mass, urinary tract problems, gynecological malformation, spinal cord tethering, perianal sepsis and meningitis. A presacral mass has been observed in 92% to 100% of patients with CS [4,7]. In pathological findings, anterior sacral meningocele and teratomas are common. In addition, presacral masses have been reported to include enteric cysts, dermoid cysts, epidermoid cysts, lipomas, leiomyosarcomas, yolk sac tumors, pelvic hamartomas and carcinoid tumors. Epidermoid cysts are rare, with a frequency of about 4% of patients with CS in adulthood [10]. We found three reports of infected epidermoid cysts associated with CS [10-12]. Two of the patients in these reports had meningitis, and the other was a two-year-old girl without meningitis. In our adult patient, CS with a presacral abscess was not combined with meningitis.

It is easy to detect a sacral defect or anomaly by pelvic X-ray. If CS is suspected, imaging of the full spine including the presacral mass is needed. CT and MRI are useful examinations for a presacral mass, as well as for spinal cord tethering. Although the reported incidence of tethered cord with CS is variable (14% to 57%) [4,7,8,13,14], it is likely to be higher than expected if more investigations are done. A tethered cord is associated with meningitis, one of the potentially lethal complications of CS. Whether meningitis occurs depends on the presence of a fistula between the spinal canal and rectum or anus. In our patient, myelography and postmyelography CT were useful, along with MRI, to identify the relationship between the presacral mass and thecal sac. If a fistula is untreated, meningitis may be encountered in the future.

Management of CS depends on the existence of a presacral mass, an anorectal malformation, and a fistula between the colon and spinal canal. Various therapeutic strategies have been reported [14-16]. Surgical treatment of a presacral mass may involve a posterior sagittal approach, a sacral laminectomy or an anterior abdominal approach when the presacral mass is too large. The posterior sagittal approach with or without anorectoplasty has been reported as the best method of treating an anorectal malformation with the simultaneous excision of the presacral mass [8]. For an anterior sacral meningocele, dural ligation of the neck of the meningocele is generally performed. In our patient, rectal stenosis was mild, and an infectious epidermoid cyst without meningitis had been diagnosed preoperatively. A two-stage operation, which consisted of drainage followed by radical excision, was successful.

The phenotypic variability and complexity of CS requires a multidisciplinary treatment. Because the Currarino triad is often missed, its diagnosis tends to be delayed. CS has a risk of serious complications resulting in morbidity and mortality. A precise preoperative diagnosis should lead to appropriate surgery and help to optimize the long-term outcome of CS.

## Conclusions

We have reported a case of CS in an adult who presented with a presacral abscess but no meningitis. Abscess drainage followed by radical surgery resulted in a successful outcome. CS consists of various phenotypes and clinical presentations, and precise multidisciplinary assessment can lead to suitable and successful treatment.

## Consent

Written informed consent was obtained from the patient for publication of this case report and any accompanying images. A copy of the written consent is available for review by the Editor-in Chief of this journal.

## Abbreviations

CS: Currarino syndrome; CT: computed tomography; MRI: magnetic resonance imaging.

## Competing interests

The authors declare that they have no competing interests.

## Author’s contributions

MS was a major contributor in writing the manuscript. NN performed the first surgery. NN and NK performed the second surgery. AY, WF, NK, HH and RI carried out the several examinations and contributed to patient management. NN followed the patient up for four years and six months and helped to draft the manuscript. All authors read and approved the final manuscript.

## References

[B1] Currarino G Coln D Votteler T Triad of anorectal, sacral, and presacral anomalies AJR Am J Roentgenol 1981 137 395 398 10.2214/ajr.137.2.395 6789651

[B2] Ross AJ Ruiz-Perez V Wang Y Hagan DM Scherer S Lynch SA Lindsay S Custard E Belloni E Wilson DI Wadey R Goodman F Orstavik KH Monclair T Robson S Reardon W Burn J Scambler P Strachan T A homeobox gene, HLXB9, is the major locus for dominantly inherited sacral agenesis Nat Genet 1998 20 358 361 10.1038/3828 9843207

[B3] Belloni E Martucciello G Verderio D Ponti E Seri M Jasonni V Torre M Ferrari M Tsui LC Scherer SW Involvement of the *HLXB9* homeobox gene in Currarino syndrome Am J Hum Genet 2000 66 312 319 10.1086/302723 10631160 PMC1288336

[B4] Lynch SA Wang Y Strachan T Burn J Lindsay S Autosomal dominant sacral agenesis: Currarino syndrome J Med Genet 2000 37 561 566 10.1136/jmg.37.8.561 10922380 PMC1734652

[B5] Berghauser Pont LM Dirven CM Dammers R Currarino's triad diagnosed in an adult woman Eur Spine J 2012 21 Suppl 4 S569 S572 22526704 10.1007/s00586-012-2311-2 PMC3369045

[B6] Kochling J Karbasiyan M Reis A Spectrum of mutations and genotype-phenotype analysis in Currarino syndrome Eur J Hum Genet 2001 9 599 605 10.1038/sj.ejhg.5200683 11528505

[B7] Urioste M Garcia-Andrade Mdel C Valle L Robledo M Gonzalez-Palacios F Mendez R Ferreiros J Nuno J Benitez J Malignant degeneration of presacral teratoma in the Currarino anomaly Am J Med Genet A 2004 128A 299 304 10.1002/ajmg.a.30028 15216552

[B8] Isik N Elmaci I Gokben B Balak N Tosyali N Currarino triad: surgical management and follow-up results of four [correction of three] cases Pediatr Neurosurg 2010 46 110 119 10.1159/000319007 20664237

[B9] Kim AY Yoo SY Kim JH Eo H Jeon TY Currarino syndrome: variable imaging features in three siblings with *HLXB9* gene mutation Clin Imaging 2013 37 398 402 10.1016/j.clinimag.2012.05.007 23466002

[B10] Haga Y Cho H Shinoda S Masuzawa T Recurrent meningitis associated with complete Currarino triad in an adult: case report Neurol Med Chir (Tokyo) 2003 43 505 508 10.2176/nmc.43.505 14620204

[B11] Shamoto H Yoshida Y Shirane R Yoshimoto T Anterior sacral meningocele completely occupied by an epidermoid tumor Childs Nerv Syst 1999 15 209 211 10.1007/s003810050372 10361973

[B12] Kansal R Mahore A Dange N Kukreja S Epidermoid cyst inside anterior sacral meningocele in an adult patient of Currarino syndrome manifesting with meningitis Turk Neurosurg 2012 22 659 661 23015348 10.5137/1019-5149.JTN.3985-10.1

[B13] Lee SC Chun YS Jung SE Park KW Kim WK Currarino triad: anorectal malformation, sacral bony abnormality, and presacral mass–a review of 11 cases J Pediatr Surg 1997 32 58 61 10.1016/S0022-3468(97)90094-4 9021570

[B14] Emans PJ van Aalst J van Heurn EL Marcelis C Kootstra G Beets-Tan RG Vles JS Beuls EA The Currarino triad: neurosurgical considerations Neurosurgery 2006 58 924 929 10.1227/01.NEU.0000209945.87233.6A 16639328

[B15] Tani S Okuda Y Abe T Surgical strategy for anterior sacral meningocele two case report Neurol Med Chir (Tokyo) 2003 43 204 209 10.2176/nmc.43.204 12760501

[B16] Martucciello G Torre M Belloni E Lerone M Pini Prato A Cama A Jasonni V Currarino syndrome: proposal of a diagnostic and therapeutic protocol J Pediatr Surg 2004 39 1305 1311 10.1016/j.jpedsurg.2004.05.003 15359381

